# Robust prognostic model based on immune infiltration‐related genes and clinical information in ovarian cancer

**DOI:** 10.1111/jcmm.17360

**Published:** 2022-06-23

**Authors:** Xi Zhang, Weikaixin Kong, Miaomiao Gao, Weiran Huang, Chao Peng, Zhuo Huang, Zhengwei Xie, Hongyan Guo

**Affiliations:** ^1^ 66482 Department of Obstetrics and Gynecology Peking University Third Hospital Beijing China; ^2^ 3835 Institute for Molecular Medicine Finland (FIMM) University of Helsinki Helsinki Finland; ^3^ Institute Sanqu Technology (Hangzhou) Co., Ltd. Hangzhou China; ^4^ Department of Pharmacology School of Basic Medical Sciences Peking University Beijing China; ^5^ 33133 Department of Molecular and Cellular Pharmacology School of Pharmaceutical Sciences Peking University Health Science Center Beijing China; ^6^ 33133 Peking University International Cancer Institute and Department of Pharmacology School of Basic Medical Sciences Peking University Health Science Center Beijing China

**Keywords:** immune infiltration, nomogram, Ovarian cancer, prognosis

## Abstract

Immune infiltration of ovarian cancer (OV) is a critical factor in determining patient's prognosis. Using data from TCGA and GTEx database combined with WGCNA and ESTIMATE methods, 46 genes related to OV occurrence and immune infiltration were identified. Lasso and multivariate Cox regression were applied to define a prognostic score (IGCI score) based on 3 immune genes and 3 types of clinical information. The IGCI score has been verified by K‐M curves, ROC curves and C‐index on test set. In test set, IGCI score (C‐index = 0.630) is significantly better than AJCC stage (C‐index = 0.541, *p* < 0.05) and CIN25 (C‐index = 0.571, *p* < 0.05). In addition, we identified key mutations to analyse prognosis of patients and the process related to immunity. Chi‐squared tests revealed that 6 mutations are significantly (*p* < 0.05) related to immune infiltration: BRCA1, ZNF462, VWF, RBAK, RB1 and ADGRV1. According to mutation survival analysis, we found 5 key mutations significantly related to patient prognosis (*p* < 0.05): CSMD3, FLG2, HMCN1, TOP2A and TRRAP. RB1 and CSMD3 mutations had small p‐value (*p* < 0.1) in both chi‐squared tests and survival analysis. The drug sensitivity analysis of key mutation showed when RB1 mutation occurs, the efficacy of six anti‐tumour drugs has changed significantly (*p* < 0.05).

## 1 BACKGROUND

Ovarian cancer is one of the most lethal gynaecological cancers, with an overall 5‐year survival rate of 48%, and is in the top five cancer types associated with death in women.^1^ Due to the unobtrusive nature of the symptoms, nearly 75% of patients present at an advanced stage, leading to a 5‐year survival rate of only 29% in the advanced stage. The first‐line therapy is tumour‐debulking surgery followed by platinum‐based chemotherapy; however, recurrence occurs in nearly 75% of patients.^2^ Moreover, there is no confirmed effective drug for patients following platinum‐resistant recurrence. Although recent clinical trials have shown that poly ADP‐ribose polymerase (PARP) inhibitors extend progression‐free survival (PFS) of patients with the BRCA1/2 mutation,^3^, ^4^ homogenous repair deficiency (HRD),^5^ or platinum‐sensitive recurrence regardless of BRCA mutation,^6^ the overall survival (OS) rate is still low.

Immunotherapy is highlighted in the search for new strategies for maintenance therapy in OV. Nonetheless, both active immunotherapy (e.g. ovarian cancer vaccine) and positive immunotherapy (e.g. adoptive T‐cell therapy) have been studied, although the response rates are not ideal.^7^ To identify novel drug targets and select patients that respond effectively to immunotherapy, it is essential to establish methods to predict the basic immune status of patients. The first step in this process is to analyse differentially expressed genes (DEGs) and immune‐related pathways associated with prognosis. Due to the variety of gene clusters identified in different types of statistical analysis, it is necessary to select an appropriate scoring strategy in relation to tumour immunity. The tumour microenvironment, which consists of immune cells, tumour cells and other components,^8^ is a promising target for the exploration of novel biomarkers. ESTIMATE is an algorithm designed by Yoshihara et al. to calculate an immune score based on differential gene signatures between stromal cells and tumour cells.^9^ This tool has been used to identify microenvironment‐related markers in several solid tumours.^10^, ^11^, ^12^

In the present study, we combined the ESTIMATE algorithm with other bioinformatic analysis tools to build an immuno‐microenvironment‐related prognosis model (IGCI score) for OV. Two mutations RB1 and CSMD3, which are closely related to immune invasion and prognosis, were identified in OV and found that in patients with RB1 mutation, the sensitivity of some drugs has changed.

## 2 MATERIALS AND METHODS

### 2.1 Data processing

‘Fragments per kilobase of exon model per million reads mapped’ (FPKM) standardized RNA sequencing data (including 379 ovarian cancer samples) and single‐nucleotide polymorphism (SNP) data (including 436 ovarian cancer samples) were obtained from TCGA (The Cancer Genome Atlas, https://portal.gdc.cancer.gov) database. Due to the lack of normal tissue samples, we downloaded the RNA sequencing data of 88 normal ovarian tissues from GTEx^13^ (The Genotype‐Tissue Expression, http://commonfund.nih.gov/GTEx/) database and performed FPKM standardization. When combining expression data obtained from TCGA, the duplicate expression of a same gene was processed to get average value as the final expression value using the ‘avereps’ function in limma package in R.

### 2.2 Screening of differentially expressed genes

DEGs between cancerous tissues and normal tissues may contain key information about disease development, so we then try to screen out the DEGs. Genes with expression were less than 0.3 in all samples were removed firstly. Then, the screening process for DEGs used the Wilcoxon signed rank test.^14^ Genes with a false discovery rate (FDR) <0.05 and |log_2_ fold change| > 1 were identified as DEGs.

### 2.3 Weighted gene correlation network analysis (WGNCA) of DEGs

WGCNA was applied to analyse DEGs in an attempt to find key gene modules and key genes related to the development of ovarian cancer.^15^ Research shows that the gene regulatory network in the organism obeys the basic structure of the scale‐free network. To achieve this goal, the correlation coefficients between genes were weighted as follows:

$$\alpha_{\mathrm{ij}}={\left|\text{cor}\left(i,j\right)\right|}^{\beta }.$$

A topological overlap matrix (TOM) was then constructed, and the distance between genes was defined by considering other genes related to these two genes. Dynamic clustering methods were used to determine the final gene modules. Genes clustering within the same module often have similar functions. Correlation analysis was performed between the first principal component of the gene modules and the tumour phenotypes (for discrete variables, 0 represents no occurrence and 1 represents occurrence), and we obtained the gene modules closely related to the occurrence of cancer. The parameters in this process were: MaxBlocksize = 7000, deepSplit = 2, minModuleSize = 40 and mergeCutHeight = 0.30. To obtain the key genes in the key modules, we obtained the module membership (MM) value and gene significance (GS) value for each gene, where MM is the Pearson correlation coefficient of gene expression and the first principal component of the module and GS is the Pearson correlation coefficient of gene expression and the cancer phenotype. Genes with large MM and GS values are generally considered to play key roles in the occurrence of disease; therefore, in this study, we assumed that those genes with MM and GS values in the upper quartile of the module are key genes in the development of ovarian cancer. The WGCNA was performed using the ‘WGCNA’ package (1.67) in R.^16^

### 2.4 DEGs related to immune infiltration

The ESTIMATE algorithm^9^ is a method of gene set analysis to evaluate the purity of tumour tissue. The ESTIMATE algorithm first performs whole‐genome sequencing data on known immune cells and tumour cells and then performs screening of DEGs. Such DEGs are selected as the background. After that, other tumour tissue sequencing data can be analysed by GSEA^17^ in this genetic background, and the score based on the degree of enrichment (ImmuneScore) can be used to evaluate the immune cell content in this tumour tissue. The StromalScore is calculated via a similar process. In this study, the ESTIMATE algorithm was used to calculate the StromalScore and ImmuneScore values in all tumour samples to clarify the degree of immune infiltration in samples. According to the median ImmuneScore, tumour samples were divided into high‐ and low‐score groups. DEGs related to ImmuneScore between low‐score and high‐score group were identified using the criteria: FDR <0.05 and | log2 fold change | > 1; DEGs related to StromalScore were identified in the same way. The final DEGs identified from the intersection of these two groups of DEGs were considered to be key genes related to immunity during the development of ovarian cancer. These genes and key genes from WGCNA were used in the construction of subsequent patient prognosis models. The ESTIMATE algorithm was applied using the ‘estimate’ package (1.0.13) in R.

### 2.5 Survival analysis and SNP analysis

The genes located at the intersection of the DEGs obtained by WGCNA and the ESTIMATE were regarded as being closely related to disease development and immune processes. We use patient clinical information and DEGs to build a prognostic model. We excluded samples that lacked clinical or gene expression information in the TCGA database, and ultimately obtained 258 patient data. We group patients according to 1:1 ratio randomly. The training data set has 130 samples and the test data set has 128 samples. We then built a prognostic model using the training set. Lasso regression^18^ was used to eliminate collinearity between different factors, with 10‐fold cross‐validation performed 1000 times. The penalty coefficient lambda selection criterion was used to obtain the smallest partial likelihood deviance. Multivariate Cox proportional hazards regression analysis^19^ was then used to build an effective prognostic model. The variable selection method is the forward–backward selection method. By adding weight to factors, we obtained the risk score for each patient according to the following formula:

$$\text{Risk}\;\;\text{core}=\sum_{i=1}^{N}{\text{Value}}_{i}\times \beta_{i},$$

where β is the coefficient of the factor in the Cox regression model, and *Valuei* is the factor level. We divided patients into high‐ and low‐risk groups according to the median risk score in the training set, and plotted the Kaplan–Meier survival curves using a log‐rank test in the training and test set. In addition, we predicted the survival of patients 1, 3 and 5 years after the onset of disease and plotted receiver operating characteristic (ROC) curves. In order to make our prognostic model more practical, we have established the corresponding nomogram and calculated the corresponding C‐index and calibration curves in the training and test data sets. We compared the prognostic results of AJCC stage and CIN25^20^ with our results to demonstrate the rationality of our model. The ‘glmnet’ (4.1–3), ‘survival’ (3.2–13), ‘survminer’ (0.4.9), ‘caret’ (6.0–90), ‘survivalROC’ (1.0.3), ‘rms’ (6.2–0) and ‘GenVisR’ (1.26.0) packages in R were used in these analyses.

Then, we performed a survival analysis in relation to the presence or absence of SNPs. After excluding patients lacking SNP data, mRNA data or clinical data, data of 272 patients from the TCGA database were analysed. We performed survival analysis on all genes with mutations identified in at least 15 patients using log‐rank tests.^21^ In addition, we also performed chi‐squared tests on the SNPs and ImmuneScore groups of patients to identify the key gene mutation related to the immune process of ovarian cancer. The mutations of no less than 5 patients were included in the chi‐squared test.

The SNPs with *P*‐value of less than 0.1 in both survival analysis and SNP analysis can be regarded as critical SNPs in ovarian cancer. We used the Genomics of Drug Sensitivity in Cancer (GDSC, https://www.cancerrxgene.org/) database for drug sensitivity analysis of key SNPs.

### 2.6 Functional and pathway enrichment analysis

Gene ontology (GO) analysis^22^ is used to identify the GO terms of enriched genes when the background of the genes and the species being studied are clear. In the absence of enrichment results for this group of genes, they should conform to the hypergeometric distribution. Kyoto Encyclopedia of Genes and Genomes (KEGG) is a utility database resource for genomic sequencing and other high‐throughput experimental technologies generated from large molecular datasets (https://www.kegg.jp/). For the genes of interest, we also performed enrichment analysis in the KEGG database to identify the key gene regulatory pathways. We focused on terms that were significantly enriched in GO and KEGG, and ranked *p*‐values from small to large. In the GO analysis, we identified the top five gene terms (*p* < 0.05). In the KEGG analysis, we identified the top three pathways (all *p* < 0.05). The ‘clusterProfiler’ (4.0.5), ‘org.HS.eg.db’ (3.13.0), ‘GOplot’ (1.0.2) and ‘digest’ (0.6.27) packages in R were used in these analyses.

**FIGURE 1:**
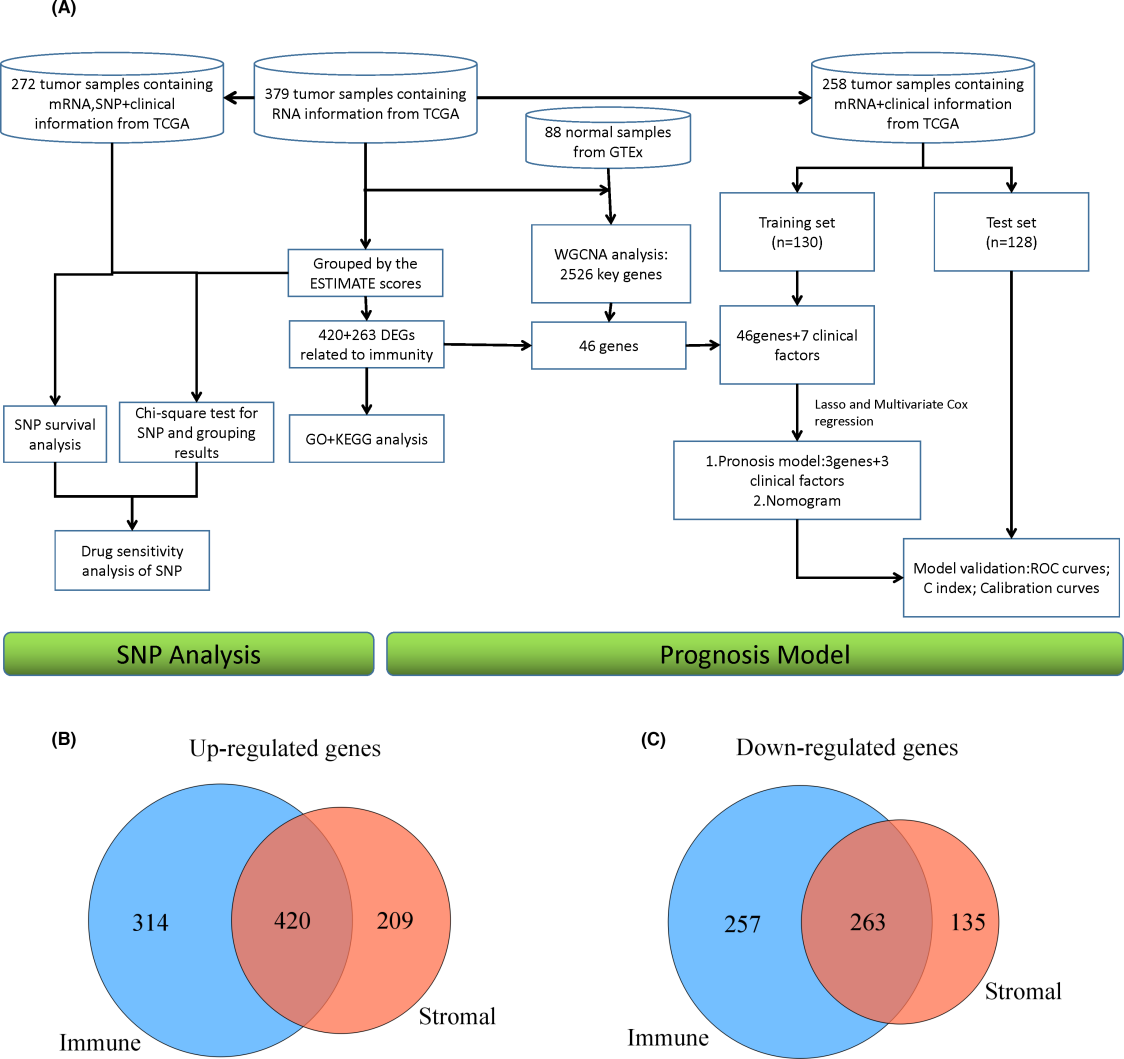
Workflow and the result of genetic intersection in the ESTIMATE algorithm. (A) Work flow chart for this study. (B) Venn diagram of up‐regulated genes in StromalScore and up‐regulated genes in ImmuneScore. (C) Venn diagram of down‐regulated genes in StromalScore and down‐regulated genes in ImmuneScore

The overall workflow of this study is shown in Figure 1A.

## 3 RESULTS

### 3.1 Identification of DEGs in ovarian cancer process and immune invasion process

We aimed to identify DEGs that are closely related to the occurrence of ovarian cancer and the immune infiltration process. Compared with 88 normal samples, 2908 genes were up‐regulated and 3162 genes were down‐regulated in 379 tumour samples (Figure S1A). These 6070 (2908 + 3162) genes could be regarded as closely related to the occurrence of OV and were used in subsequent WGCNA analysis.

It is generally believed that the degree of tumour immune infiltration is closely related to the content of stromal cells and immune cells in tumour samples. Compared with the low StromalScore group, a total of 734 genes were up‐regulated and 398 genes were down‐regulated in the high StromalScore group (Figure S1B). Compared with the low ImmuneScore group, 629 genes were up‐regulated and 520 genes were down‐regulated genes in the high ImmuneScore group (Figure S1C). From the intersection of StromalScore and ImmuneScore, the up‐ (420 genes, Figure 1B) and down‐regulated genes (263 genes, Figure 1C) were identified, and these genes can be regarded as genes that play a key role in the progress of tumour immune infiltration.

### 3.2 Results of DEGs GO and KEGG gene enrichment analysis

**FIGURE 2:**
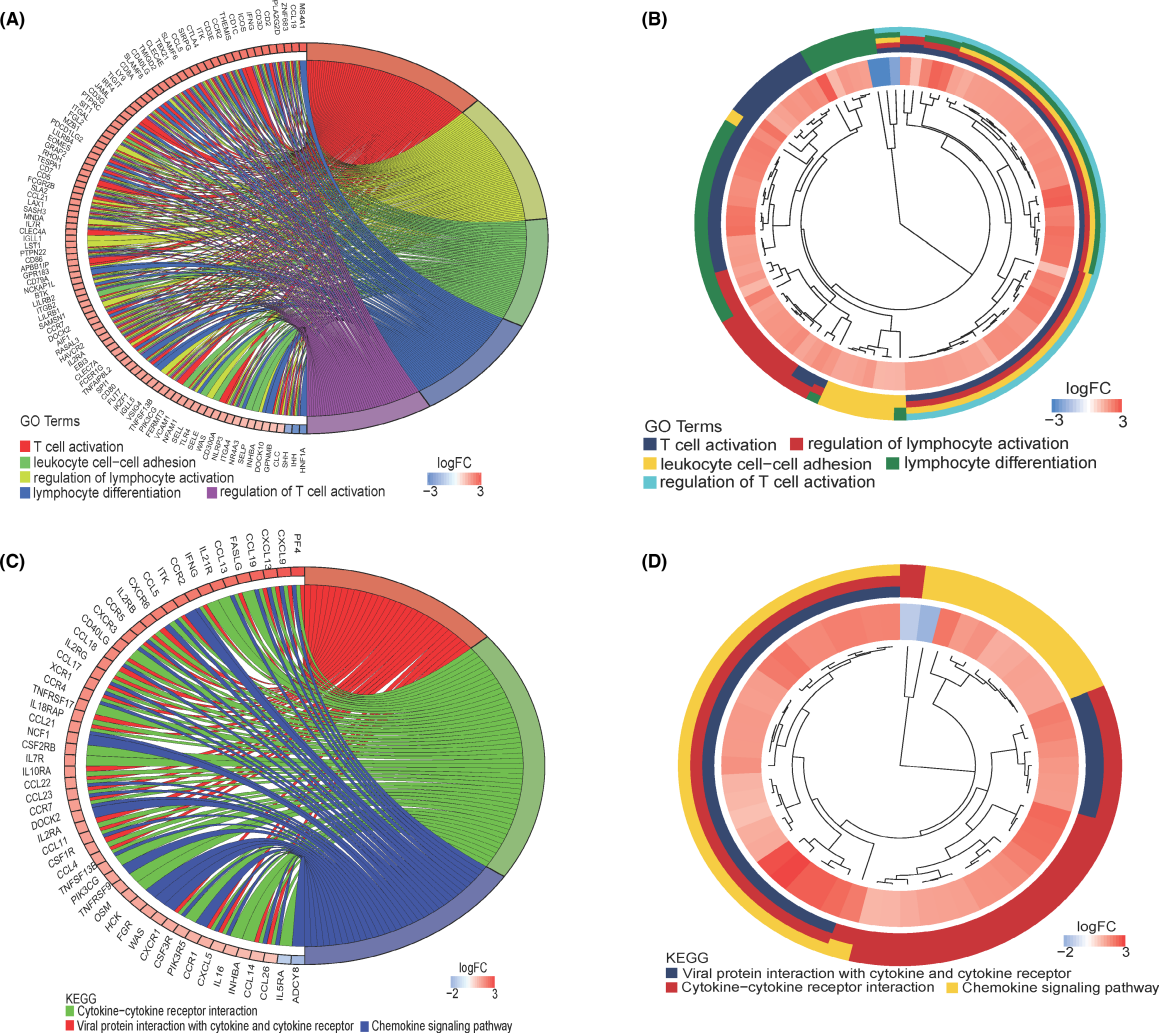
GO and KEGG gene pathway enrichment analyses of the DEGs identified using the ESTIMATE algorithm. (A) Circle graph of GO analysis of DEGs. The different colours on the right half of the circle graph represent different GO terms, and the shades of colour on the left half of the circle graph represent the log_2_(fold change) in gene expression. (B) Differential gene GO analysis clustering results. The middle circle represents the log_2_(fold change) change in expression. The different colours in the outer circle represent different GO terms. (C) KEGG pathway analysis circle diagram. Different colours represent different KEGG pathways. (D) Clustering results of KEGG pathway analysis. The different colours in the outer circles represent different KEGG pathways

To verify the method of grouping according to the scores assigned by the ESTIMATE algorithm was indeed applicable to our investigations and achieve a better understanding of the roles of the identified 683 DEGs (420 + 263) in ovarian cancer, GO and KEGG pathway enrichment analysis was performed. The top five GO terms were T‐cell activation; regulation of lymphocyte activation; leukocyte cell–cell adhesion; lymphocyte differentiation; and regulation of T‐cell activation. These terms showed that the DEGs obtained according to the ESTIMATE algorithm are closely related to the immune process in tumour tissues and confirmed the effectiveness of the StromalScore and ImmuneScore (Figure 2A,B). It can be observed that many genes are involved in these five GO terms. CCL19, ZNF683, PLA2G2D and CD2 genes not only had the largest fold change in expression, but were also in all of the top five GO terms, indicating that these genes may be more critical in the immune process. The top three KEGG terms were viral protein interaction with cytokine and cytokine receptor between viral proteins and cytokines as the basis of viral infection and pathogenicity; cytokine–cytokine receptor interaction; and chemokine signalling pathway (Figure 2C,D). About 15% of human cancers can be attributed to virus infection.^23^ In addition to their association with tumour metastasis and inflammation, chemokines are also closely related to regulation of the immune system. Chemokines not only affect the migration and differentiation of lymphocytes,^24^ but also are closely related to the maturation, differentiation and functional effects of T and B lymphocytes.^25^ PF4, CXCL9, CXCL13 and CCL19 were also enriched in all of the top three KEGG pathways and had the largest fold changes on expression.

### 3.3 WGCNA of DEGs related to ovarian cancer

**FIGURE 3:**
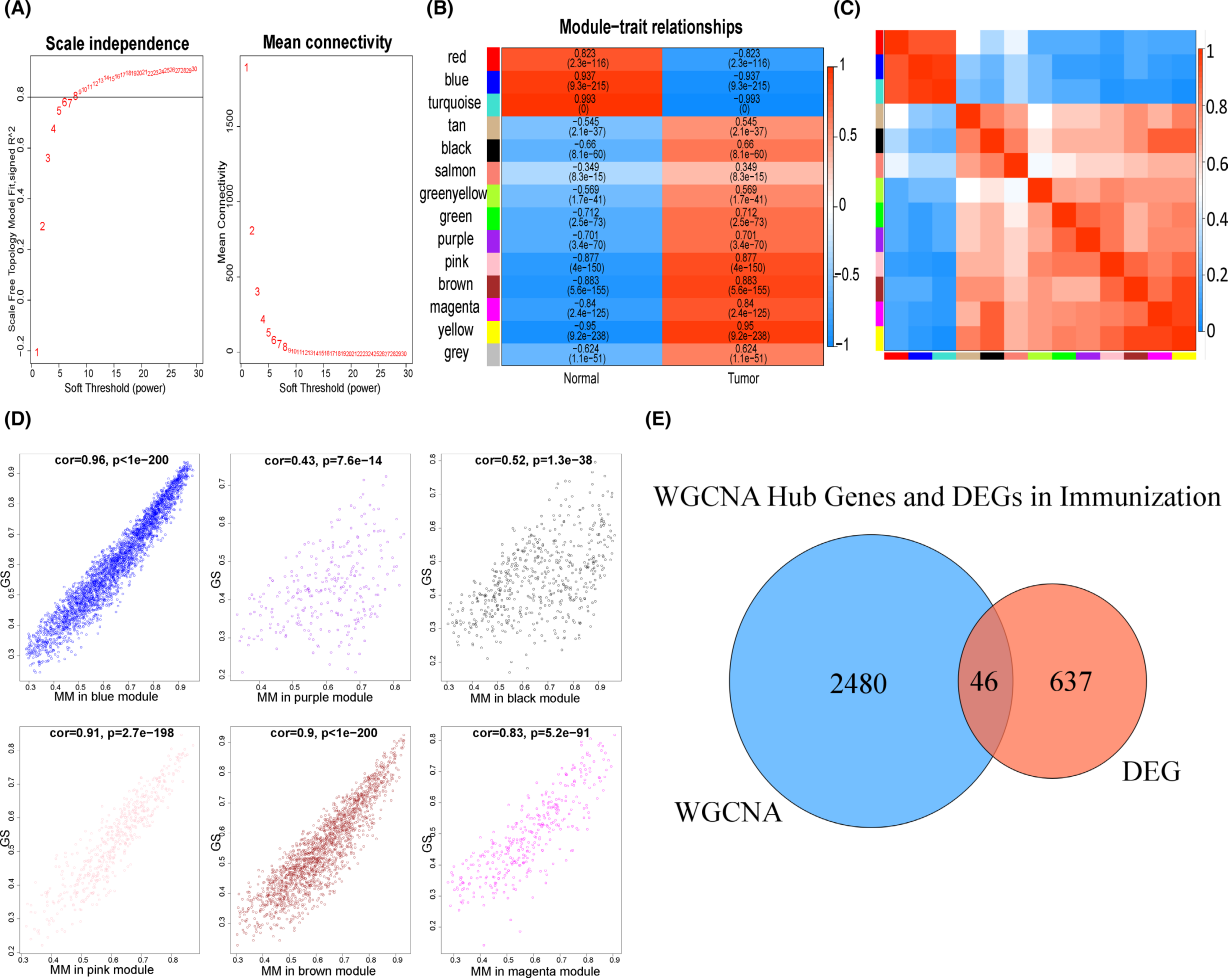
WGCNA analysis results. (A) Soft threshold selection. Left: The *y*‐axis is the *R* ^2^ value of the regression equation for the scale‐free network, and the *x*‐axis is *β*. The black line value is 0.8. Right: The *y*‐axis is the average connectivity of the network, and the *x*‐axis is *β*. (B) Module‐phenotype diagram. The size of the Pearson correlation coefficient is indicated by colour; *p*‐values are shown in parentheses. (C) Heatmap of the relationships between gene modules. The correlation between modules is measured by the Pearson correlation coefficient of the first principal component in the modules. (D) Scatter plot of gene importance in the module. The *x*‐axis is the module membership (MM) value and the *y*‐axis is the gene significance (GS) value. Correlation coefficients and *p*‐values in the subtitles indicate the linear characteristics of the scatter plot. (E) Venn diagram of the intersection of WGCNA Hub genes and DEGs by ESTIMATE algorithm

To make the connectivity of the gene regulatory network obey the power law distribution, we exponentially weighted the correlation coefficients of genes. A soft threshold (weight) of beta = 8 better meets the requirements of scale‐free networks (Figure 3A). We obtained 14 gene modules through dynamic clustering and then performed a correlation analysis between the gene modules and the occurrence of tumours (Figure 3B). Based on previous reports,^26^ we assumed that when the correlation coefficient >0.65, the module was the key gene module in the process of disease, and the hub genes were selected from these modules. As shown in Figure 3B, the red, blue, turquoise, black, green, purple, pink, grown, magenta and yellow modules had a coefficient >0.65 and were included in the subsequent analysis. In addition, the correlation analysis between modules (Figure 3C) showed that the similarity among red, blue and turquoise gene modules was high; the similarity among the brown, magenta and yellow gene modules was high; and the similarity among the tan, black and pink gene modules was high. The scatter plot of gene importance is shown in Figure 3D and Supplementary Figure 1D–F. A total of 2526 genes were obtained by selecting key genes in the upper quartile of the horizontal and vertical coordinates of these gene modules.^27^ A total of 46 genes were obtained from the intersection of these genes with the DEGs obtained using the ESITIMATE algorithm (Figure 3E). These genes were regarded as related both to the occurrence of ovarian cancer and the degree of immune infiltration.

### 3.4 The establishment of the IGCI score

The degree of immune infiltration is often closely related to the tumour recurrence and the amount of tumour stem cells. The tumour recurrence is related to the patient's final survival status. Therefore, 46 genes from the intersection of the key genes of WGCNA and the DEGs by ESTIMATE algorithm were used, and then, Lasso regression was performed on these genes and 7 clinical factors (Age; Asian, 1 means yes, 0 means not; Black, 1 means yes, 0 means not; White,1 means yes, 0 means not; Stage; Pharmaceutical. Therapy, 1 means yes, 0 means not; Radiation. Therapy, 1 means yes, 0 means not) in the training set. The summary of the patient's clinical information is shown in Table 1. The criteria of selecting clinical factors are that the ration of samples with blank values are no more than 90%. Then, to prove our random grouping of patients is reasonable, we used the *t*‐test for continuous variables and the chi‐squared test for discrete variables to compare the clinical information of the training set and the test set. As shown in Table 1, all clinical information has no significant difference between the training set and the test set (*p* > 0.05), indicating this grouping can be used in subsequent studies.

**TABLE 1:** Patient clinical baseline data

Characteristics	Train sets (*n* = 130)	Test sets (*n* = 128)	*t*/*χ* ^2^ value	*p*‐value
Age (mean ± *SD*)	59.55±11.46	59.09±11.32	0.3261	0.747
Race (%)			2.1846	0.335
White	115 (88.5)	115 (89.8)		
Black	9 (6.9)	11 (8.60)		
Asian	6 (4.6)	2 (1.6)		
Stage (%)			3.9815	0.137
I or II	13 (10.0)	7 (5.5)		
III	91 (70.0)	103 (80.5)		
IV	26 (20.0)	18 (14.0)		
Pharmaceutical (%)			0.55596	0.456
Yes	125 (96.2)	126 (98.4)		
No	5 (3.8)	2 (1.6)		
Radiation (%)			1.9711	0.16
Yes	4 (3.1)	10 (7.8)		
No	126 (96.9)	118 (92.2)		
Survival state (%)			0	1
Death	65 (50.0)	64 (50.0)		
Alive	65 (50.0)	64 (50.0)		

In lasso regression, by minimizing partial likelihood deviance, we selected the penalty coefficient lambda (Figure S2A,B), which was 0.007; the remaining 10 factors were included in the subsequent study. The levels of these 10 factors in the training set are shown in Table S1. Through Multivariate Cox proportional hazards regression analysis, we constructed a prognostic score based on immune genes and clinical information (IGCI) for patients with ovarian cancer:

ICGIscore=0.0232×Age‐0.6457×White‐3.6304×Pharmaceutical.Therapy+0.4089×FGF7‐0.1290×CCR1+0.0085×CD14.

**FIGURE 4:**
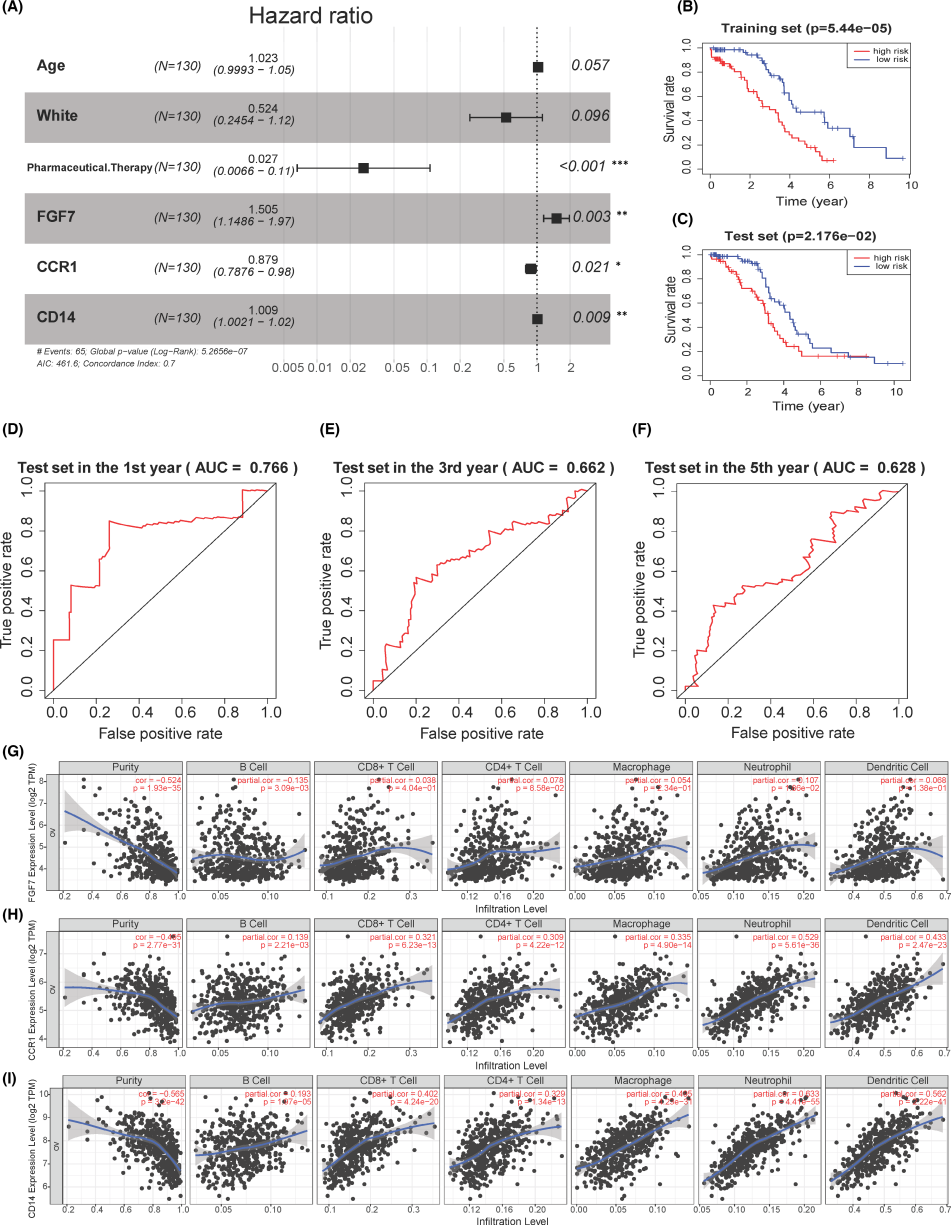
Results of the IGCI score and the results from TIMER database. (A) Forest graph for Multivariate Cox proportional hazards regression. The hazard radio and 95% confidence intervals of the 6 factors included in the model are shown, where hazard radio =e coefficient. (B) Survival analysis grouped by median IGCI score in the training set. (C) Survival analysis grouped by the same median IGCI score in the test set. (D–F) The results of predicting the 1‐, 3‐ and 5‐year survival status of patients according to the cox model (IGCI score) in the test set. The AUC value is in parentheses of the main title. The *x*‐axis is the false‐positive rate and the *y*‐axis is the true‐positive rate. (G–I) The correlation between the abundance of immune cell and the expression of (G) OR2W3, (H) RALGAPA2, (I) PTGIS in OV patients. ‘Purity’ represents the purity of the tumour cells in the sample

The high level of Age, FGF7 and CD14 was found to be disadvantageous for patients’ prognosis, while high level of White, Pharmaceutical. Therapy and CCR1 was favourable factor for patients’ prognosis. We calculated the hazard radio (HR) of each gene and the corresponding 95% confidence interval, as shown in Figure 4A. More details of the multivariate Cox proportional hazards regression are shown in Table S2.

The patient groups were subdivided according to median IGCI score in the training set. As the IGCI score gradually increased, the survival time decreased, and the proportion of patients’ deaths gradually increased in both training and test set (Figure S2C,D), which indicated the accuracy of IGCI score. In addition, analysis of patient survival status based on IGCI score groups showed significant differences (Figure 4B,C). The *p*‐value in the training set is less than 0.001 (Figure 4B), and the *p*‐value in the test set is less than 0.05 (Figure 4C). In the training set, the 1‐, 3‐ and 5‐year OS rates in the low‐risk group were 97.3% (95% CI = 91.3–100.0), 77.1% (95% CI = 65.4–90.8) and 47.0% (95% CI = 33.0–65.9); the 1‐, 3‐ and 5‐year OS rates for the high‐risk group were 90.8% (95% CI = 84.0–98.1), 46.5% (95% CI = 33.7–64.0) and 14.5% (95% CI = 6.5–32.0). In the test set, the 1‐, 3‐ and 5‐year OS rates in the low‐risk group were 97.1% (95% CI = 92.0–100.0), 78.2% (95% CI = 66.9–91.3) and 34.3% (95% CI = 21.8–54.0); the 1‐, 3‐ and 5‐year OS rates for the high‐risk group were 86.1% (95% CI = 77.1–96.3), 48.7% (95% CI = 35.4–66.8) and 16.1% (95% CI = 7.1–36.5). The IGCI score obtained was used to predict the disease status of patients at 1, 3 and 5 years. The AUC of the obtained ROC curves in test set were 0.766, 0.662 and 0.628 (Figure 4D–F). The AUC of the obtained ROC curves in training set were 0.802, 0.720 and 0.711 (Figure S3A–C). These results reflected the practical application value of the IGCI score.

TIMER database (https://cistrome.shinyapps.io/timer/) was used to explore the relationship between the genes in the IGCI score and the content of various immune cells. There was a positive correlation between FGF7 expression and the infiltration of Neutrophil cells (Cor = 0.107, *p* = 1.86e‐02), and there was a negative correlation between FGF7 expression and the infiltration of B cells (Cor = −0.135, *p* = 3.09e‐03, Figure 4G). CCR1 expression was positively associated with the infiltration of B cells (Cor = 0.139, *p* = 2.21e‐03), CD8+ T cells (Cor = 0.321, *p* = 6.23e‐13), CD4+ T cells (Cor = 0.309, *p* = 4.22e‐12), macrophage cells (Cor = 0.335, *p* = 4.90e‐14), neutrophil cells (Cor = 0.529, *p* = 5.61e‐36) and dendritic cells (Cor = 0.433, *p* = 2.47e‐23; Figure 4H). CD14 expression was positively associated with the infiltration of B cells (Cor = 0.193, *p* = 1.97e‐05), CD8+ T cells (Cor = 0.402, *p* = 4.24e‐20), CD4+ T cells (Cor = 0.329, *p* = 1.34e‐13), macrophage cells (Cor = 0.495, *p* = 4.23e‐31), neutrophil cells (Cor = 0.633, *p* = 4.41e‐55) and dendritic cells (Cor = 0.562, *p* = 2.22e‐41; Figure 4I). The above results further verify that the genes in the IGCI score are closely related to the immune infiltration process of OV and may be effective prognostic markers.

Then, to explore the relationship of factors and IGCI score, we used Wilcoxon test to see difference in a chromosomal instability score (CIN25)^20^ in low‐ and high‐risk groups. It was obvious that IGCI score was not associated with CIN25 score (Figure S4A,B), which indicated that the IGCI score is not related to chromosomal instability. IGCI score was associated with age significantly but the Spearman coefficient was limited (0.61, Figure S4C,D). This may be due to that IGCI can provide additional information beyond ageing. In addition, through chi‐squared test (Table S3), we found that IGCI is not related to pharmaceutical therapy, but this factor is significant in Cox regression.

**FIGURE 5:**
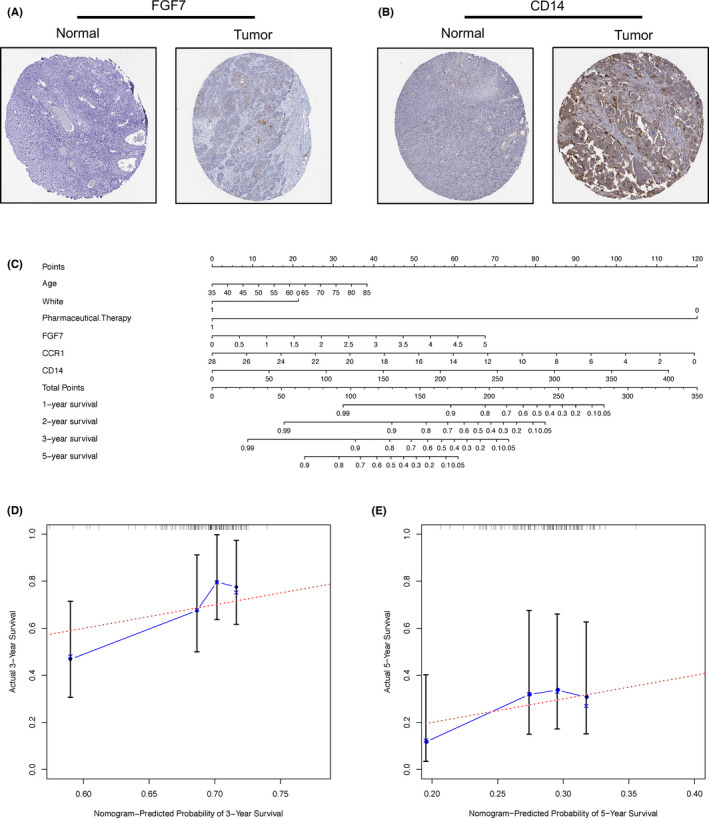
IHC results, nomogram and calibration curves. (A) IHC results of FGF gene in normal tissues and ovarian cancer tissues. (B) IHC results of CD14 gene in normal tissues and ovarian cancer tissues. (C) The nomogram for prognostic judgment. ‘Points’ is a scoring scale for each factor, and ‘total points’ is a scale for total score. Based on the total score of the patient, the 1‐, 2‐, 3‐ and 5‐ year survival rate can be inferred. In ‘Pharmaceutical. Therapy’, 1 represents treatment, 0 represents no treatment; in ‘White’ 1 represents yes, and 0 represents not. (D) Calibration curve for the predicted 3‐year survival rate in the test set. The *x*‐axis is the predicted survival rate and the *y*‐axis is the actual survival rate. The red line is the ideal result, and the error bar indicates the range of the standard error. (E) Calibration curve for the predicted 5‐year survival rate in the test set

In order to explore the content of prognostic genes at the protein level, we used data on immunohistochemistry (IHC) datasets (the Human Protein Atlas database, http://www.proteinatlas.org/) to explore the content of FGF7, CCR1 and CD14 in protein levels in ovarian cancer and control groups. The results are shown in Figure 5A,B. The corresponding sample information is shown in the Table S4. The IHC database lacks the corresponding data for the CCR1 gene. FGF7 (antibody: HPA043605) and CD14 (antibody: HPA001887) have higher protein content in the tissues of ovarian cancer patients, the staining of IHC sections is deeper, and the results of CD14 are more obvious. These two genes are disadvantages in our prognostic model. The results of IHC and the prognostic model are consistent.

To assist the clinical work of ovarian cancer, we established a nomogram based on the IGCI score (Figure 5C). To test our IGCI score, we collected popular OV signatures to perform as baseline models. At present, AJCC stage is often used to predict the prognostic status of OV patients. In addition, Carter et al.^20^ proved that a signature of chromosomal instability containing 25 genes (CIN25) can also effectively predict the prognostic status of OV patients. Zhang et al.^20^ proved the glycolysis and m5A RNA methylation processes can precisely reflect the prognosis state of OV patients; then, they developed related signatures (GRG score and m6A score), respectively, which were test in independent cohorts. In order to verify the validity of the IGCI score and the nomogram, we calculated the C‐index of the IGCI score and compared with the C‐index of the AJCC stage, CIN25, GRG score and m6A score. The results are shown in Table 2. The C‐index of the IGCI score in the training and test set were significantly higher than the results of the other scores. In addition, we have plotted the 3‐year and 5‐year survival rate calibration curves to evaluate the predictive power of the IGCI score. In the training (Figure S3D,E) and test (Figure 5D,E) set, our prediction results are close to the ideal results (red lines), and the errors are within the standard error range. This shows that the prediction results of the IGCI score are accurate.

**TABLE 2:** C‐index results for IGCI score, AJCC stage and CIN25

Method	Training set		Test set	
	C‐index (95% CI)	*p*	C‐index (95% CI)	*p*
IGCI score	0.701 (0.625,0.779)		0.630 (0.542,0.719)	
AJCC stage	0.568 (0.493,0.642)	<0.05	0.541 (0.477,0.604)	<0.05
CIN25	0.543 (0.461,0.625)	<0.05	0.571 (0.533,0.608)	<0.05

### 3.5 Genetic mutation analysis

**FIGURE 6:**
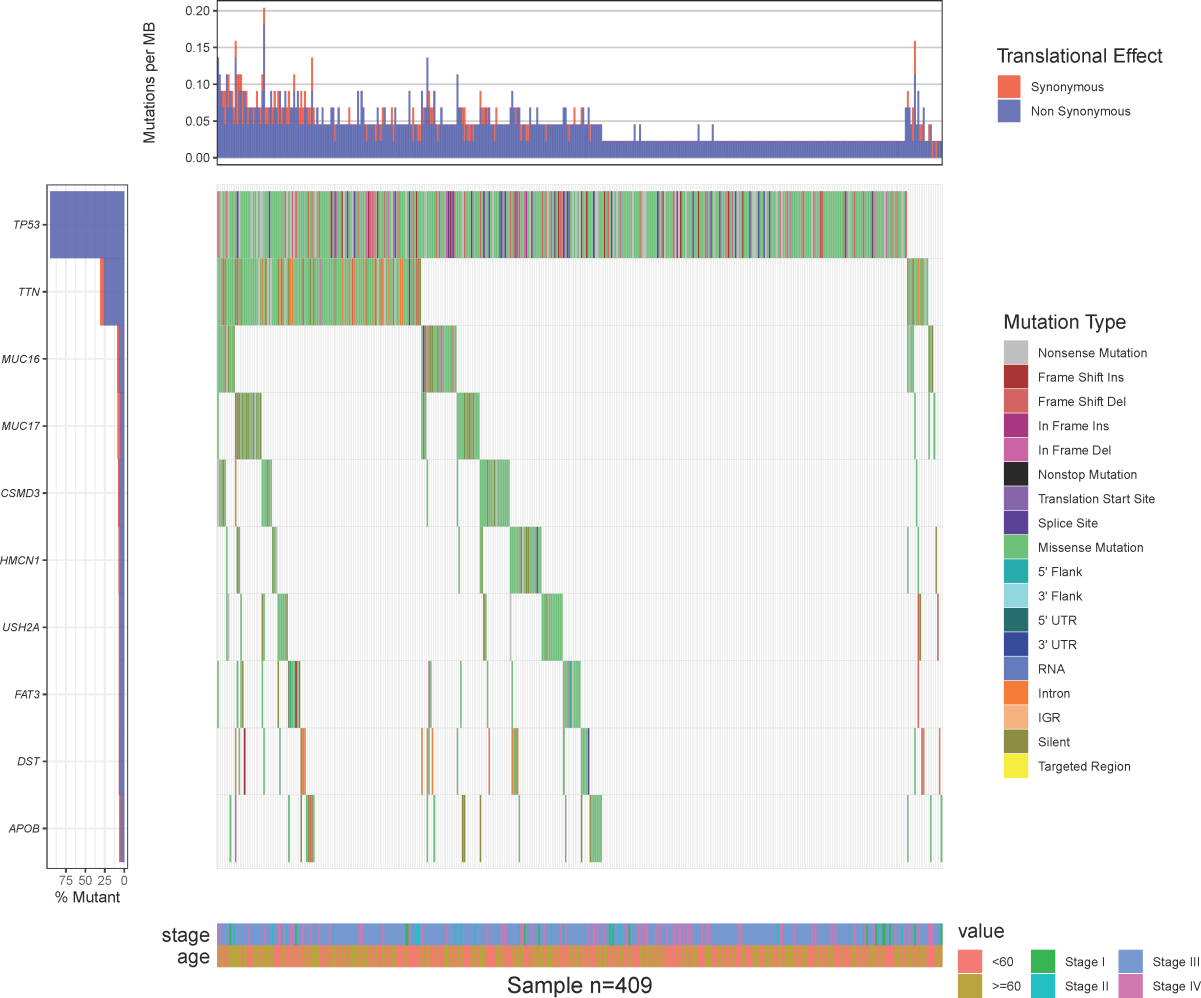
Waterfall chart for genetic mutation analysis. Each column in the figure represents a sample. The legend above shows the density of synonymous and non‐synonymous mutations in each sample, and the legend on the left shows the ratio of gene mutations in 409 samples. The legend on the right shows the type of genetic mutations and the legend below shows the clinical information of the samples

**FIGURE 7:**
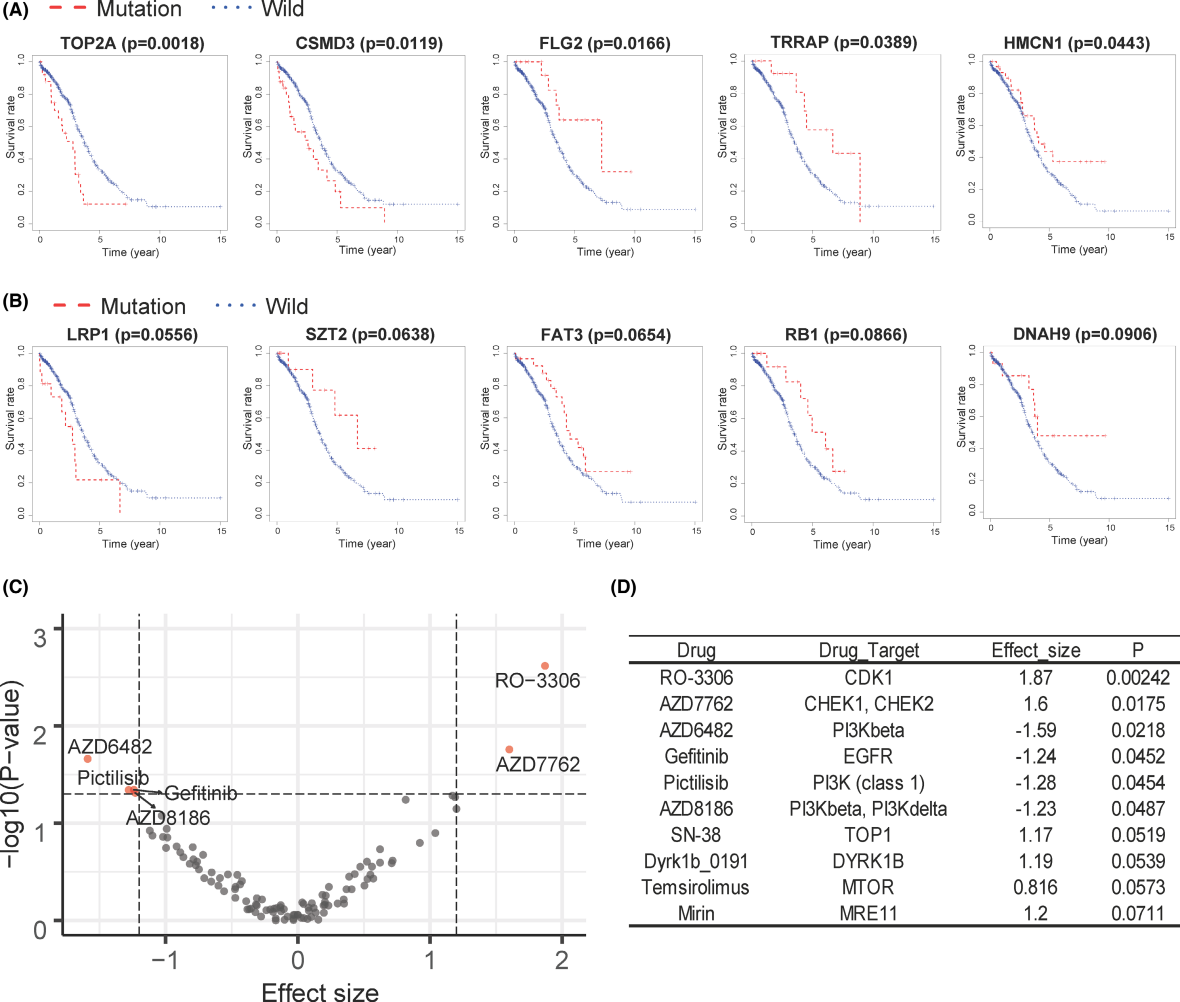
Single‐nucleotide polymorphism prognostic analysis. (A, B) The *x*‐axis is the survival time (year), the *y*‐axis is the survival rate, the red line is the sample containing the gene mutation, and the blue line is the wild‐type sample. (A) Survival plots for all gene mutations with *p* < 0.05. (B) Survival curves of all gene mutations with 0.05 < *p* < 0.1. (C) Volcano diagram of drug sensitivity analysis in OV. The red dot are the drugs whose effect changes significantly (*p* < 0.05) when RB1 is mutated. A small *x*‐axis value represents the increased sensitivity; a large *x*‐axis value represents the increased resistance. (D) The top ten drugs in the drug sensitivity analysis

To understand the types of gene mutations that are closely related to ovarian cancer, we first generated a waterfall map of the type of genetic mutation (Figure 6). From TCGA database, we obtained 409 samples with complete information for types of genetic mutations and clinical data and the top 10 genes with the most mutations were selected for display (Figure 6). The waterfall map showed no clear relationships among the stage of cancer, patient age and gene mutations (Figure 6). Compared with other genes, TP53 had a variety of mutation types, while TTN and DST were found to be more prone to mutations in the intron regions. The survival analysis of gene mutations revealed that the mutation of five genes significantly affect the prognosis of patients (Figure 7A): TOP2A (*p *= 0.0018), CSMD3 (*p *= 0.0119), FLG2 (*p *= 0.0166), TRRAP (*p *= 0.0389) and HMCN1 (*p *= 0.0443). Five genes showed marginal significance (0.05 < *p* < 0.1, Figure 7B): LRP1 (*p* = 0.0556), SZT2 (*p* = 0.0638), FAT3 (*p* = 0.0654), RB1 (*p* = 0.0866) and DNAH9 (*p* = 0.0906).

We performed a chi‐squared test (Table 3) on the gene mutations and the median ImmuneScore grouping. The mutations with *p* < 0.05 included as follows: BRCA1, RBAK, ZNF462, ADGRV1, RB1 and VWF. The mutations with 0.05 <*p* < 1 included as follows: APOB, CSMD1, DNAH3, KCNA6, COL11A1, ZNF638, AMER1, SPOCD1, SORBS2, ZNF774, LTN1, KIF3C, RANBP6, ADCY5, CSMD3 and PKHD1.

**TABLE 3:** Chi‐squared test of gene mutation and immune infiltration scores

Gene	Mutation +high score	Mutation +low score	Wild +high score	Wild +low score	*p*
BRCA1	10	2	126	134	0.0387
RBAK	0	6	136	130	0.039
ZNF462	0	6	136	130	0.039
ADGRV1	8	1	128	135	0.0419
RB1	1	8	135	128	0.0419
VWF	8	1	128	135	0.0419
APOB	11	3	125	133	0.0547
CSMD1	3	11	133	125	0.0547
DNAH3	9	2	127	134	0.0647
KCNA6	0	5	136	131	0.0709
COL11A1	5	0	131	136	0.0709
ZNF638	0	5	136	131	0.0709
AMER1	0	5	136	131	0.0709
SPOCD1	0	5	136	131	0.0709
SORBS2	5	0	131	136	0.0709
ZNF774	5	0	131	136	0.0709
LTN1	0	5	136	131	0.0709
KIF3C	5	0	131	136	0.0709
RANBP6	0	5	136	131	0.0709
ADCY5	0	5	136	131	0.0709
CSMD3	19	9	117	127	0.0725
PKHD1	10	3	126	133	0.0881

The RB1 and CSMD3 mutation had small *p*‐value in both the prognostic process (RB1: *p* = 0.0866; CSMD3: *p* = 0.0119) and the chi‐squared test (RB1: *p* = 0.0419; CSMD3: *p* = 0.0725). This shows that RB1 and RSMD3 mutations may play an important role in OV patients. In order to explore the clinical value of RB1 and RSMD3 mutations, we used the GDSC database for drug sensitivity analysis. RSMD3 mutation was not included in the GDSC database. The result of the RB1 mutation was shown in Figure 7C,D. The efficacy of AZD6482, Pictilisib, AZD8186 and Gefitinib in RB1 mutant samples was more significant (*p* < 0.05), while the efficacy of AZD7762 and RO‐3306 in RB1 mutant samples decreased (*p* < 0.05). The above results indicate that the occurrence of RB1 mutation may make the patient's response to drugs change greatly. Compared with the wild type, the prognosis of patients with RB1 mutation is better (Figure 7B). On this basis, the use of drugs with enhanced efficacy for these patients may get better treatment results.

## 4 DISCUSSION

### 4.1 Key factors in the IGCI score

In the IGCI score, age is a poor prognostic factor, which is closely related to the decline in physical function of the elderly. In addition, though ‘Pharmaceutical. Therapy’ factor cannot be identified at the diagnosis state, the clinician can use this nomogram with this factor to predict the survival rate change after these two kinds of treatments.

The FGF7 gene has the largest absolute coefficient (0.4089, Table S2) compared with other genes in the IGCI score, indicating that the change in FGF7 gene expression has the greatest effect on the prognosis of patients. In humans, 22 FGFs have been identified (FGF1––FGF22), and most of their sequences are conserved.^28^ The main function of FGFs is to facilitate receptor ligand interaction. FGF7 is a growth factor in the FGFs family and functions as a mitogen. Furthermore, inhibiting the expression of FGF7 significantly reduces the division of tumour tissues.^29^ There are currently many anticancer drugs that target the FGF‐FGFR pathway. For example, FIIN‐2 and FIIN‐3 covalently bind to cysteine 486 in the P‐loop of FGFR,^30^ irreversibly blocking the activation of FGFR and the phosphorylation of the downstream signalling molecule ERK1/2. These drugs are more commonly used in the chemotherapy of lung cancer although it can be speculated that FGF7‐related regulation may also be a potential target in ovarian cancer. The absolute value of the coefficient of FGF7 in the IGCI score was the largest, indicating that FGF7 can also be used as a prognostic predictor of ovarian cancer.

When it comes to the CCR1, tumour cells secrete chemokines, which act on stromal cells through CCR1 to induce chemotaxis, and cooperate with stromal cells, promote the invasion process and transfer to the blood circulation or lymphatic system.^31^ Chemokines participate in the formation, invasion and metastasis of tumours such as epithelial cell carcinoma, squamous cell carcinoma and mesenchymal cell carcinoma.^32^ Interestingly, in the IGCI score, the coefficient of CCR1 is −0.1290, indicating that this is a favourable factor for prognosis. This may be the result of the body's own negative feedback regulation, but it is still valid information for the patient's prognosis, or maybe CCR1 has regulatory functions in the body that we have not yet understood.

The CD14 antigen is a 365‐amino acid phosphatidylinositol‐binding glycoprotein, which is mainly expressed on monocytes and macrophage membranes in body tissues.^33^ In tumour tissues, macrophages mainly infiltrate the pericarcinoma and cancer interstitial tissues.^34^ The detection rate of macrophages represents the immune status of local tumour tissues. Therefore, CD14 can effectively reflect the level of immune infiltration in patients with ovarian cancer. In our prognostic model, the HR of CD14 is =0.879 <1 (Figure 4A), which is a favourable factor for prognosis. Cancer patients with a large degree of immune infiltration often have better prognosis,^35^ which is consistent with the results of our model.

### 4.2 Key genes in the gene mutation analysis

Six mutations are of great significance between high ImmuneScore grouping and low ImmuneScore grouping according to chi‐squared test: BRCA1, ZNF462, VWF, RBAK, RB1 and ADGRV1. According to SNP survival analysis, we found the mutation of five genes significantly affect the prognosis of patients: CSMD3, FLG2, HMCN1, TOP2A and TRRAP. Among these genes, BRCA1, RB1, RBAK and CSMD3 are tumour suppressors, while TRRAP functions as an oncogene. In addition to tumour suppressors and promotors in tumour cells, these genes might also in other cells in tumour microenvironment. Tumour microenvironment cell components are composed of tumour cells, immune cells and fibroblasts. Previous studies have revealed the function of CSMD3 in dendritic cells and regulation of HMCN1 in fibroblasts,^36^ suggesting these genes may regulate various components of the ovarian cancer microenvironment.

Tumours with BRCA1 mutations are defective in repairing DNA damage thorough the homologousre combination pathway. Consequently, BRCA1 represents a potential therapeutic target in gynaecological cancer, breast cancer,^37^, ^38^, ^39^ prostate cancer^40^ and other cancers.^41^ PARPi have shown effect in various cancers with BRCA1/2 mutations.^42^ PARPi have been confirmed to be beneficial to patients with ovarian cancer independently of the state of BRCA,^43^ indicating that further studies are warranted to identify novel targets and biomarkers. TOP2A, also known as TOP2, encodes a DNA topoisomerase that controls the topologic structure of double‐stranded DNA during by binding to the 5'‐ends of double‐strand breaks (DSB). DSB promotes the transient formation of the TOP2‐cleavage complex and gene transcription and are then repaired by homologous repair (HR) and/or nonhomologous end‐joining (NHEJ). Defects in this process lead to carcinogenic genotoxicity. BRCA‐1, which contributes to HR in S/G2‐phase cells and NHEJ in G1‐phase cells, negatively regulates carcinogenesis, especially in breast cancer and ovarian cancer.^44^

TRRAP is a subunit of histone acetyltransferase and a key cofactor for c‐Myc, which is an oncogenic DNA‐binding transcription activator. By recruitment of TRRAP, c‐Myc activates RNA polymerases I and III to control ribosome biogenesis and cell growth. It has been confirmed that TRRAP positively regulates the accumulation of mutant p53 in lymphoma, and TRRAP inhibition by histone deacetylases decreases mutant p53 levels.^45^ In addition, TRRAP depletion leads to down‐regulation of TOP2A, which is consistent with our results and indicates that the association between these two genes is worthy of exploration in ovarian cancer.

The SNP of RB1 is closely related to immune process (Table 3). This evidence strongly illustrates the potential of RB1 as an immune and prognostic marker in ovarian cancer. A previously reported model indicated that RB1 functions as an essential tumour suppressor, which physically interacts with RBAK.^46^ In ovarian cancer, the concurrent inactivation of P53 and RB1 is adequate forcarcinogenesis,^47^ and in addition, RB1 may promote chemotherapy resistance.^48^ Based on comprehensive studies on the BRCA state and DNA repair, a model has been proposed to illustrate the relationship between P53 and RB1, as well as homologous repair deficiency.^49^ RBAK was computationally predicted as a downstream target of miR‐155 in lymphoma,^50^ although the function of RBAK in solid tumours remains poorly understood. Our analysis revealed differential regulation of RBAK and RB1 in patients, which implies the importance of interactions between these two genes in ovarian cancer and their potential as drug targets. Further studies are required to confirm the involvement of RBAK in this process following its interaction with RB1.

CSMD3 is a member of CSMD gene family. The non‐synonymous mutation of CSMD3 has been identified in familial colorectal cancer but not in healthy controls.^51^ Whole‐exome sequencing has revealed that CSMD3 is the second most frequently mutated gene after TP53 in non‐small cell lung carcinoma, and loss of CSMD3 causes proliferation of airway epithelial cells.^52^ Additionally, CRISPR/Cas9‐mediated knockout of CSMD3 inhibits the death of PDX tumour cells, which also suggests that CSMD3 is an important tumour suppressor.^53^

HMCN1, which is a conserved extracellular member of the immunoglobulin superfamily, manages epithelial cell attachments. As a cell polarity regulatory gene, HMCN1 is significantly up‐regulated in gastric carcinoma.^54^ Moreover, mutation of HMCN1 is associated with metastasis in breast cancer.^55^ In ovarian cancer, HMCN1 may promote invasiveness by regulating cancer‐associated fibroblasts.^36^ Newly generated fibrocytes act as a ‘wall’ that prevents the entry of immune cells into the ovarian cancer site.

As a plasma glycoprotein, von Willebrand factor (vWF) mediates the attachment of platelets confronted with damaged endothelium.^56^ A large population‐based study demonstrated the association between coagulation, inflammation and survival of cancer patients,^57^ indicating that increased mortality in cancer survivors is dependent on high vWF levels. Our data also show that vWF expression is related to immune scores and patient survival, suggesting vWF as a novel biomarker of the basic immune state and predicts long‐term survival in cancer patients.

For the first time, we report the immune‐related mutations of ZNF462, ADGRV1 and FLG2 in ovarian cancer. As a zinc‐finger protein, ZNF462 may take part in embryonic development and is associated with neurodevelopmental abnormalities.^58^ ZNF462 may be the target of miR‐210,^59^ which could be induced by hypoxia‐inducible factor‐1alpha in pancreatic cancer. ADGRV1 is one of the biallelic pathogenic identification markers of Usher syndrome,^60^ which is characterized by congenital bilateral sensorineural hearing loss.^61^ The deficiency of FLG2 causes defective adhesion between cornified cells, which leads to peeling skin syndrome.^62^ However, the roles of these two genes in cancer remain unclear, and further studies are required.

## 5 CONCLUSION

In the present study, data from TCGA and the GTEx database were combined with the ESTIMATE algorithm to identify 46 genes closely related to OV occurrence and immune infiltration process. Using genes and clinical information together, we established the IGCI score containing six essential factors. The IGCI score and the corresponding nomogram have been effectively verified by the ROC curves, C‐index and calibration curves on the test set. The prediction ability of the IGCI score is better than AJCC stage (*p* < 0.05) and CIN25 (*p* < 0.05). In addition, by analysing, the gene mutations related to the process of ovarian cancer, the gene mutations that are closely related to the patient's prognosis and the degree of immune infiltration were identified. We conducted drug sensitivity analysis on key gene mutation, which provides a reference for subsequent research.

## AUTHOR CONTRIBUTIONS

**Xi Zhang:** Conceptualization (equal); Data curation (lead); Formal analysis (lead); Validation (equal); Writing – original draft (lead); Writing – review & editing (lead). **Weikaixin Kong:** Conceptualization (lead); Data curation (lead); Writing – original draft (equal); Writing – review & editing (equal). **Miaomiao Gao:** Data curation (equal); Resources (equal); Visualization (equal). **Weiran Huang:** Formal analysis (equal). **Chao Peng:** Investigation (supporting); Validation (supporting). **Zhuo Huang:** Funding acquisition (lead); Supervision (lead); Writing – original draft (supporting); Writing – review & editing (supporting). **Zhengwei Xie:** Funding acquisition (lead); Methodology (lead); Supervision (lead). **Hongyan Guo:** Conceptualization (lead); Funding acquisition (lead); Investigation (supporting); Methodology (lead); Supervision (lead); Writing – original draft (supporting); Writing – review & editing (supporting).

## CONFLICT OF INTEREST

The authors declare that there are no conflict of interests.

## Supporting information

Fig S1

Fig S2

Fig S3

Fig S4

Table S1

Table S2

Table S3

Table S4

## Data Availability

The data involved in this study can be obtained through the TCGA (https://portal.gdc.cancer.gov/) and GTEx (https://www.gtexportal.org/home/index.html) databases.

## References

[1] Siegel RL , Miller KD , Jemal A . Cancer statistics, 2020. CA Cancer J Clin. 2020;70(1):7‐30. 31912902 10.3322/caac.21590

[2] Li Y , McGrail DJ , Xu J , et al. MERIT: systematic analysis and characterization of mutational effect on RNA interactome topology. Hepatology. 2019;70(2):532‐546. 30153342 10.1002/hep.30242 PMC6538468

[3] Kaufman B , Shapira‐Frommer R , Schmutzler RK , et al. Olaparib monotherapy in patients with advanced cancer and a germline BRCA1/2 mutation. J Clin Oncol. 2015;33(3):244‐250. 25366685 10.1200/JCO.2014.56.2728 PMC6057749

[4] Stockler MR , Hilpert F , Friedlander M , et al. Patient‐reported outcome results from the open‐label phase III AURELIA trial evaluating bevacizumab‐containing therapy for platinum‐resistant ovarian cancer. J Clin Oncol. 2014;32(13):1309‐1316. 24687829 10.1200/JCO.2013.51.4240 PMC4876313

[5] Coleman RL , Oza AM , Lorusso D , et al. Rucaparib maintenance treatment for recurrent ovarian carcinoma after response to platinum therapy (ARIEL3): a randomised, double‐blind, placebo‐controlled, phase 3 trial. Lancet. 2017;390(10106):1949‐1961. 28916367 10.1016/S0140-6736(17)32440-6 PMC5901715

[6] Mirza MR , Monk BJ , Herrstedt J , et al. Niraparib maintenance therapy in platinum‐sensitive, recurrent ovarian cancer. N Engl J Med. 2016;375(22):2154‐2164. 27717299 10.1056/NEJMoa1611310

[7] Kandalaft LE , Odunsi K , Coukos G . Immunotherapy in ovarian cancer: are we there yet? J Clin Oncol 2019;37(27):2460‐2471. 31403857 10.1200/JCO.19.00508

[8] Binnewies M , Roberts EW , Kersten K , et al. Understanding the tumor immune microenvironment (TIME) for effective therapy. Nat Med. 2018;24(5):541‐550. 29686425 10.1038/s41591-018-0014-x PMC5998822

[9] Yoshihara K , Shahmoradgoli M , Martínez E , et al. Inferring tumour purity and stromal and immune cell admixture from expression data. Nat Commun. 2013;4:11. 10.1038/ncomms3612 PMC3826632 24113773

[10] Mahal BA , Alshalalfa M , Zhao SG , et al. Genomic and clinical characterization of stromal infiltration markers in prostate cancer. Cancer. 2020;126(7):1407‐1412. 31905251 10.1002/cncr.32688 PMC7332205

[11] Stewart PA , Welsh EA , Slebos RJC , et al. Proteogenomic landscape of squamous cell lung cancer. Nat Commun. 2019;10(1):3578. 31395880 10.1038/s41467-019-11452-x PMC6687710

[12] Zhao J , Chen AX , Gartrell RD , et al. Immune and genomic correlates of response to anti‐PD‐1 immunotherapy in glioblastoma. Nat Med. 2019;25(3):462‐469. 30742119 10.1038/s41591-019-0349-y PMC6810613

[13] Lonsdale J , Thomas J , Salvatore M , et al. The Genotype‐Tissue Expression (GTEx) project. Nat Genet. 2013;45(6):580‐585. 23715323 10.1038/ng.2653 PMC4010069

[14] Rosner B , Glynn RJ , Lee MLT . The Wilcoxon signed rank test for paired comparisons of clustered data. Biometrics. 2006;62(1):185‐192. 16542245 10.1111/j.1541-0420.2005.00389.x

[15] Botía JA , Vandrovcova J , Forabosco P , et al. An additional k‐means clustering step improves the biological features of WGCNA gene co‐expression networks. BMC Syst Biol. 2017;11:16. 28403906 10.1186/s12918-017-0420-6 PMC5389000

[16] Langfelder P , Horvath S . WGCNA: an R package for weighted correlation network analysis. BMC Bioinformatics. 2008;9:13. 19114008 10.1186/1471-2105-9-559 PMC2631488

[17] Shi J , Walker MG . Gene set enrichment analysis (GSEA) for interpreting gene expression profiles. Curr Bioinform. 2007;2(2):133‐137.

[18] Lu YM , Zhou Y , Qu WB , Deng MH , Zhang CG . A Lasso regression model for the construction of microRNA‐target regulatory networks. Bioinformatics. 2011;27(17):2406‐2413. 21743061 10.1093/bioinformatics/btr410

[19] Gui J , Li HZ . Penalized Cox regression analysis in the high‐dimensional and low‐sample size settings, with applications to microarray gene expression data. Bioinformatics. 2005;21(13):3001‐3008. 15814556 10.1093/bioinformatics/bti422

[20] Carter SL , Eklund AC , Kohane IS , Harris LN , Szallasi Z . A signature of chromosomal instability inferred from gene expression profiles predicts clinical outcome in multiple human cancers. Nat Genet. 2006;38(9):1043‐1048. 16921376 10.1038/ng1861

[21] Mizukami A , Matsue Y , Naruse Y , et al. Kaplan‐Meier survival analysis and Cox regression analyses regarding right ventricular septal pacing: data from Japanese pacemaker cohort. Data Brief. 2016;8:1303‐1307. 27570808 10.1016/j.dib.2016.07.058 PMC4990661

[22] Kim HI , Kim JH , Park YJ . Transcriptome and gene ontology (GO) enrichment analysis reveals genes involved in biotin metabolism that affect l‐lysine production in *Corynebacterium glutamicum* . Int J Mol Sci. 2016;17(3):12. 10.3390/ijms17030353 PMC4813214 27005618

[23] Yeo W , Zee B , Zhong S , et al. Comprehensive analysis of risk factors associating with Hepatitis B virus (HBV) reactivation in cancer patients undergoing cytotoxic chemotherapy. Br J Cancer. 2004;90(7):1306‐1311. 15054446 10.1038/sj.bjc.6601699 PMC2409681

[24] Kunkel EJ , Butcher EC . Chemokines and the tissue‐specific migration of lymphocytes. Immunity. 2002;16(1):1‐4. 11825560 10.1016/s1074-7613(01)00261-8

[25] Bourges D , Wang CH , Chevaleyre C , Salmon H . T and IgA B lymphocytes of the pharyngeal and palatine tonsils: differential expression of adhesion molecules and chemokines. Scand J Immunol. 2004;60(4):338‐350. 15379858 10.1111/j.0300-9475.2004.01479.x

[26] Li J , Liu C , Chen YI , et al. Tumor characterization in breast cancer identifies immune‐relevant gene signatures associated with prognosis. Front Genet. 2019;10:10. 31781173 10.3389/fgene.2019.01119 PMC6861325

[27] Zhou Z , Cheng Y , Jiang Y , et al. Ten hub genes associated with progression and prognosis of pancreatic carcinoma identified by co‐expression analysis. Int J Biol Sci. 2018;14(2):124‐136. 29483831 10.7150/ijbs.22619 PMC5821034

[28] Trueb B . Biology of FGFRL1, the fifth fibroblast growth factor receptor. Cell Mol Life Sci. 2011;68(6):951‐964. 21080029 10.1007/s00018-010-0576-3 PMC11115071

[29] Wesche J , Haglund K , Haugsten EM . Fibroblast growth factors and their receptors in cancer. Biochem J. 2011;437:199‐213. 21711248 10.1042/BJ20101603

[30] Tan LI , Wang J , Tanizaki J , et al. Development of covalent inhibitors that can overcome resistance to first‐generation FGFR kinase inhibitors. Proc Natl Acad Sci USA. 2014;111(45):E4869‐E4877. 25349422 10.1073/pnas.1403438111 PMC4234547

[31] Kitamura T , Fujishita T , Loetscher P , et al. Inactivation of chemokine (C‐C motif) receptor 1 (CCR1) suppresses colon cancer liver metastasis by blocking accumulation of immature myeloid cells in a mouse model. Proc Natl Acad Sci USA. 2010;107(29):13063‐13068. 20616008 10.1073/pnas.1002372107 PMC2919974

[32] Wu X , Fan J , Wang X , et al. Downregulation of CCR1 inhibits human hepatocellular carcinoma cell invasion. Biochem Biophys Res Commun. 2007;355(4):866‐871. 17336272 10.1016/j.bbrc.2007.01.199

[33] Wright SD , Ramos RA , Tobias PS , Ulevitch RJ , Mathison JC . CD14, a receptor for complexes of lipopolysaccharide (LPS) And LPS Binding‐Protein. Science. 1990;249(4975):1431‐1433. 1698311 10.1126/science.1698311

[34] Yakirevich E , Maroun L , Cohen O , Izhak OB , Rennert G , Resnick MB . Apoptosis, proliferation, and Fas (APO‐I, CD95)/Fas ligand expression in medullary carcinoma of the breast. Journal of Pathology. 2000;192(2):166‐173. 11004692 10.1002/1096-9896(2000)9999:9999<::AID-PATH689>3.0.CO;2-A

[35] Gopalakrishnan V , Helmink BA , Spencer CN , Reuben A , Wargo JA . The influence of the gut microbiome on cancer, immunity, and cancer immunotherapy. Cancer Cell. 2018;33(4):570‐580. 29634945 10.1016/j.ccell.2018.03.015 PMC6529202

[36] Liu CL , Pan HW , Torng PL , Fan MH , Mao TL . SRPX and HMCN1 regulate cancer‐associated fibroblasts to promote the invasiveness of ovarian carcinoma. Oncol Rep. 2019;42(6):2706‐2715. 31638245 10.3892/or.2019.7379

[37] Tutt A , Tovey H , Cheang MCU , et al. Carboplatin in BRCA1/2‐mutated and triple‐negative breast cancer BRCAness subgroups: the TNT Trial. Nat Med. 2018;24(5):628‐637. 29713086 10.1038/s41591-018-0009-7 PMC6372067

[38] Robson M , Im S‐A , Senkus E , et al. Olaparib for metastatic breast cancer in patients with a germline BRCA mutation. N Engl J Med. 2017;377(6):523‐533. 28578601 10.1056/NEJMoa1706450

[39] Litton JK , Rugo HS , Ettl J , et al. Talazoparib in patients with advanced breast cancer and a germline BRCA mutation. N Engl J Med. 2018;379(8):753‐763. 30110579 10.1056/NEJMoa1802905 PMC10600918

[40] Pritchard CC , Mateo J , Walsh MF , et al. Inherited DNA‐repair gene mutations in men with metastatic prostate cancer. N Engl J Med. 2016;375(5):443‐453. 27433846 10.1056/NEJMoa1603144 PMC4986616

[41] Mateo J , Carreira S , Sandhu S , et al. DNA‐repair defects and olaparib in metastatic prostate cancer. N Engl J Med. 2015;373(18):1697‐1708. 26510020 10.1056/NEJMoa1506859 PMC5228595

[42] Lord CJ , Ashworth A . PARP inhibitors: synthetic lethality in the clinic. Science (New York, NY). 2017;355(6330):1152‐1158. 10.1126/science.aam7344 PMC6175050 28302823

[43] Shen J , Zhao W , Ju Z , et al. PARPi triggers the STING‐dependent immune response and enhances the therapeutic efficacy of immune checkpoint blockade independent of BRCAness. Can Res. 2019;79(2):311‐319. 10.1158/0008-5472.CAN-18-1003 PMC6588002 30482774

[44] Sasanuma H , Tsuda M , Morimoto S , et al. BRCA1 ensures genome integrity by eliminating estrogen‐induced pathological topoisomerase II‐DNA complexes. Proc Natl Acad Sci USA. 2018;115(45):E10642‐E10651. 30352856 10.1073/pnas.1803177115 PMC6233096

[45] Jethwa A , Słabicki M , Hüllein J , et al. TRRAP is essential for regulating the accumulation of mutant and wild‐type p53 in lymphoma. Blood. 2018;131(25):2789‐2802. 29653964 10.1182/blood-2017-09-806679

[46] Skapek SX , Jansen D , Wei T‐F , et al. Cloning and characterization of a novel Kruppel‐associated box family transcriptional repressor that interacts with the retinoblastoma gene product, RB. J Biol Chem. 2000;275(10):7212‐7223. 10702291 10.1074/jbc.275.10.7212

[47] Flesken‐Nikitin A , Choi KC , Eng JP , Shmidt EN , Nikitin AY . Induction of carcinogenesis by concurrent inactivation of p53 and Rb1 in the mouse ovarian surface epithelium. Can Res. 2003;63(13):3459‐3463. 12839925

[48] El Shamieh S , Saleh F , Assaad S , Farhat F . Next‐generation sequencing reveals mutations in RB1, CDK4 and TP53 that may promote chemo‐resistance to palbociclib in ovarian cancer. Drug Metab Pers Therap. 2019;34(2). doi:10.1515/dmpt-2018-0027 31145688

[49] Peng G , Mills GB . Surviving ovarian cancer: an affair between defective DNA repair and RB1. Clin Cancer Res. 2018;24(3):508‐510. 29191971 10.1158/1078-0432.CCR-17-3022 PMC5905329

[50] Mehrotra M , Medeiros LJ , Luthra R , et al. Identification of putative pathogenic microRNA and its downstream targets in anaplastic lymphoma kinase‐negative anaplastic large cell lymphoma. Hum Pathol. 2014;45(10):1995‐2005. 25128227 10.1016/j.humpath.2014.06.012

[51] Gylfe AE , Sirkiä J , Ahlsten M , et al. Somatic mutations and germline sequence variants in patients with familial colorectal cancer. Int J Cancer. 2010;127(12):2974‐2980. 21351276 10.1002/ijc.25529

[52] Liu P , Morrison C , Wang L , et al. Identification of somatic mutations in non‐small cell lung carcinomas using whole‐exome sequencing. Carcinogenesis. 2012;33(7):1270‐1276. 22510280 10.1093/carcin/bgs148 PMC3499051

[53] Zhang P , Kang B , Xie G , et al. Genomic sequencing and editing revealed the GRM8 signaling pathway as potential therapeutic targets of squamous cell lung cancer. Cancer Lett. 2019;442:53‐67. 30391781 10.1016/j.canlet.2018.10.035

[54] Chen C , Shi C , Huang X , et al. Molecular profiles and metastasis markers in Chinese patients with gastric carcinoma. Sci Rep. 2019;9(1):13995. 31570735 10.1038/s41598-019-50171-7 PMC6769015

[55] Saravia CH , Flores C , Schwarz LJ , et al. Patterns of mutation enrichment in metastatic triple‐negative breast cancer. Clin Med Insights Oncol. 2019;13:1179554919868482. 10.1177/1179554919868482 PMC6689917 31447598

[56] Haberichter SL . von Willebrand factor propeptide: biology and clinical utility. Blood. 2015;126(15):1753‐1761. 26215113 10.1182/blood-2015-04-512731 PMC4600015

[57] Panova‐Noeva M , Schulz A , Arnold N , et al. Coagulation and inflammation in long‐term cancer survivors: results from the adult population. J Thromb Haemost. 2018;16(4):699‐708. 29431889 10.1111/jth.13975

[58] Weiss K , Wigby K , Fannemel M , et al. Haploinsufficiency of ZNF462 is associated with craniofacial anomalies, corpus callosum dysgenesis, ptosis, and developmental delay. Eur J Hum Genet. 2017;25(8):946‐951. 28513610 10.1038/ejhg.2017.86 PMC5567153

[59] Chen W‐Y , Liu W‐J , Zhao Y‐P , et al. Induction, modulation and potential targets of miR‐210 in pancreatic cancer cells. Hepatobiliary Pancreat Dis Int. 2012;11(3):319‐324. 22672828 10.1016/s1499-3872(12)60168-4

[60] Jouret G , Poirsier C , Spodenkiewicz M , et al. Genetics of Usher syndrome: new insights from a meta‐analysis. Otol Neurotol. 2019;40(1):121‐129. 30531642 10.1097/MAO.0000000000002054

[61] Lentz J , Keats B . Usher syndrome type II. In: Adam MP, Ardinger HH, Pagon RA, et al.,. In: Adam MP Ardinger HH Pagon RA *GeneReviews(^®^)*. Seattle (WA): University of Washington, Seattle; 1993.

[62] Alfares A , Al‐Khenaizan S , Al MF . Peeling skin syndrome associated with novel variant in FLG2 gene. Am J Med Genet A. 2017;173(12):3201‐3204. 28884927 10.1002/ajmg.a.38468

