# Supplementary figures and images for: Robust prognostic model based on immune infiltration‐related genes and clinical information in ovarian cancer
Source: J Cell Mol Med. 2022 Jun 23;26(13):3659–74. doi: 10.1111/jcmm.17360 (PMC9258710; doi:10.1111/jcmm.17360)

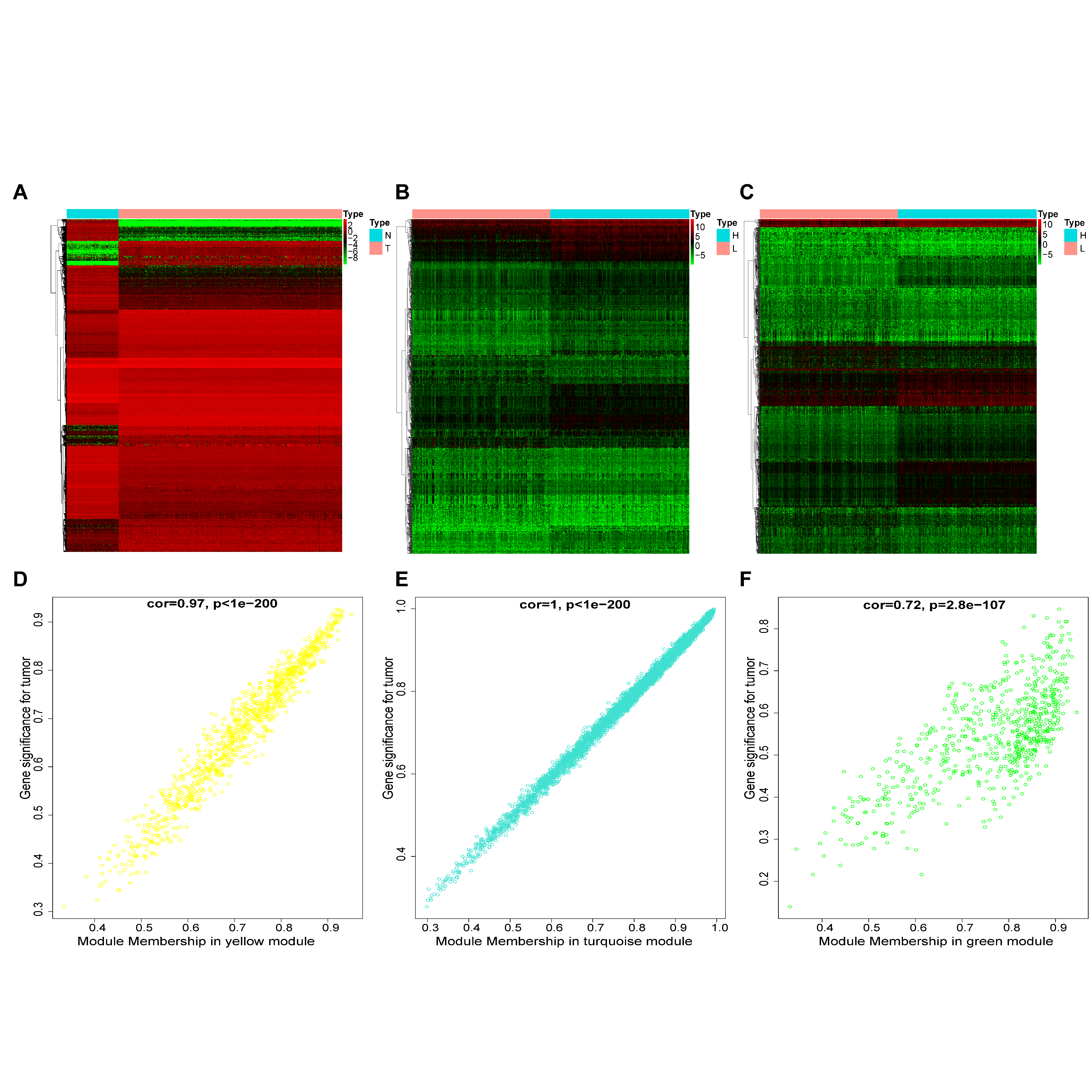

Supplement: Supplementary file 1 — Fig S1 [file JCMM-26-3659-s008.tiff]

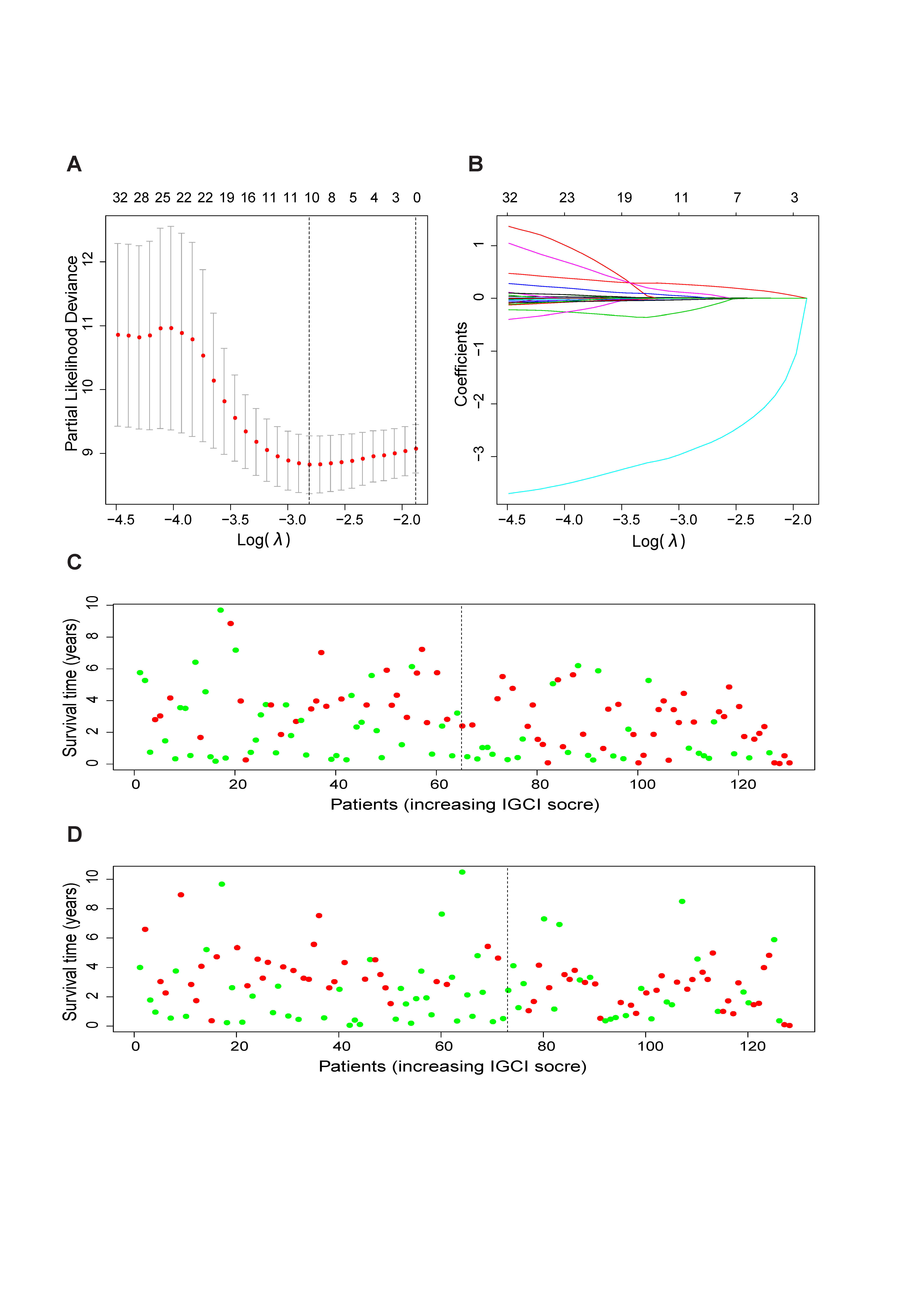

Supplement: Supplementary file 2 — Fig S2 [file JCMM-26-3659-s003.tiff]

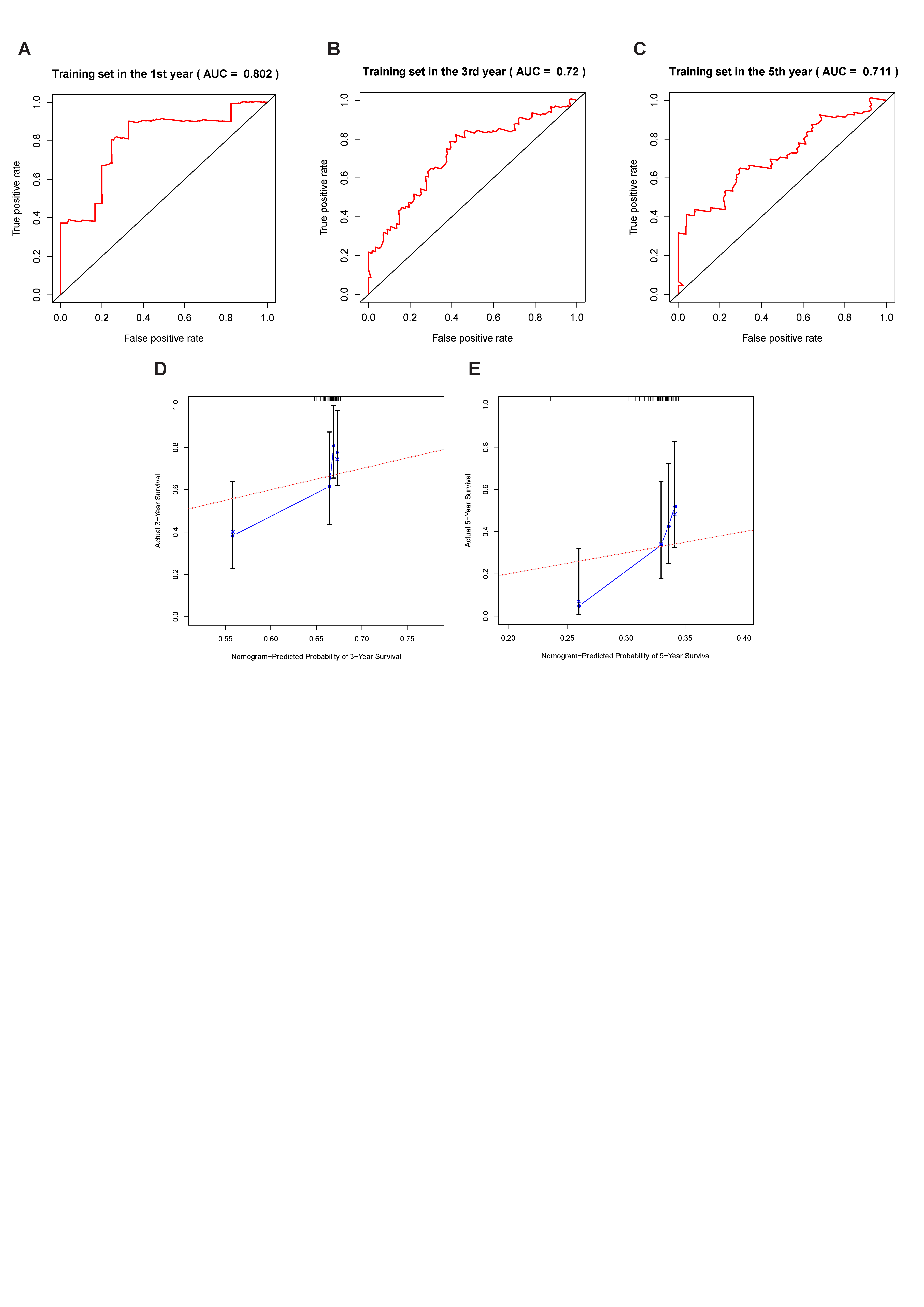

Supplement: Supplementary file 3 — Fig S3 [file JCMM-26-3659-s002.tiff]
